# Supplementary material for: The influence of social support, physical activity, general psychological distress, and demographic characteristics on self-reported health status among women in Iran
Source: PLoS One. 2026 Feb 23;21(2):e0343015. doi: 10.1371/journal.pone.0343015 (PMC12928592; doi:10.1371/journal.pone.0343015)
Supplement: Attachment — Submitted filename: Editors Comments Response..docx [file pone.0343015.s003.docx]

**Dear editorial board of *Journal of Plos One***

# Thanks for providing the comments of the respectful reviewer to us. We tried to revise the manuscript, titled “The Influence of Social Support, Physical Activity, psychological problems, and Demographic Characteristics on Self-Reported Health Status among Women” based on the comments and respond it in the following table. Revisions has been shown as highlight in the manuscript. Hope the revisions are satisfactory now. However, we welcome any further constructive comments if required.

**Dr. Hadi Darvishigilan**

**Corresponding author**

| **Comments** | **Response** |
| --- | --- |
| 1. Foremost, although an ethics code (IR.KUMS.REC.1403.078) is provided, there is no IRCT registration number. Please clarify it. | Thank you for your comments. This study is not a clinical trial study, so it does not have an IRCT code.  This study is the result of a research project conducted with code 4030146 from Kermanshah University of Medical Sciences. |
| 2. The statistical analysis needs to be revised and clarified. The logistic regression model lacks information on variable selection, control for confounders, assumption testing (normality, multiple collinearity), and goodness-of-fit assessment. Confidence intervals for several odds ratios are missing, and the SRH duality needs to be justified. | We appreciate your valuable comments. The section on Statistical analysis has been thoroughly revised to provide greater clarity and methodological transparency. |
| 3. Please confirm obviously that the study population and dataset are completely independent of the population used in your previously published article in Scientific Reports (2025; DOI: 10.1038/s41598-024-79835-9) | The mentioned study (DOI: 10.1038/s41598-024-79835-9) is the result of a research project with the code 4020003, which was conducted in early 2023 with the participation of 495 people aged 18 to 75 (men and women). This is while the submitted manuscript is the result of another research project with the code 4030146, which was conducted in 2024. The participants were 350 women aged 18 to 75, and the data from this study is completely independent of the study published in the journal Scientific Reports. |
| 4. Lastly, the manuscript requires significant improvements in language and formatting, including the correction of grammatical errors, consistent verb tense usage, and proper reference formatting. | The manuscript was improved in language and formatting. |
