# Supplementary material for: The influence of social support, physical activity, general psychological distress, and demographic characteristics on self-reported health status among women in Iran
Source: PLoS One. 2026 Feb 23;21(2):e0343015. doi: 10.1371/journal.pone.0343015 (PMC12928592; doi:10.1371/journal.pone.0343015)
Supplement: Attachment — Submitted filename: Reviewers Comments Response..docx [file pone.0343015.s004.docx]

**Dear editorial board of *Journal of Plos One***

# Thanks for providing the comments of the respectful reviewer to us. We tried to revise the manuscript, titled “The Influence of Social Support, Physical Activity, General Psychological Distress, and Demographic Characteristics on Self-Reported Health Status among Women in Iran” based on the comments and respond it in the following table. Revisions has been shown as highlight in the manuscript. Hope the revisions are satisfactory now. However, we welcome any further constructive comments if required.

**Dr. Hadi Darvishigilan**

**Corresponding author**

| **Comments** | **Response** |
| --- | --- |
| **Reviewer 1** | |
| 1. Overall, as a reader of the paper, I felt that the paper is very well written and have important content for professionals in the relevant fields. Congratulations to the authors for their wonderful work. | Many thanks for your attention and times. |
| **Reviewer 2** | |
| 1. We suggest specifying the context of the study in the title and abstract (in Iran). | Many thanks for your comments. It was conducted.  Page 1, line 2 and Page 2, line 41. |
| **Introduction** | |
| 1. Overall, the introduction is well written and clear. The authors described the relevance of the topic. However, the existence of 5 concepts (4 exposures and 1 outcome) in the study may make things hard to assimilate. Therefore, please specify why the authors chose to assess these four exposures in relation to self-reported health. | Many thanks for your comments. Explanations for the reason for choosing four exposures in relation to self-reported health were added to the end of the introduction.  Page 4, lines 106-109 and Page 5, lines 110-112. |
| 2. Line 99-101: The idea expression is complex to understand. Please avoid using too many linking adverbs consecutively (additionally, consequently, etc.) | Many thanks for your comments. It was revised. Page 4, lines 96-101. |
| 3. Line 109: Provide a reference to research conducted that reveals that studies concerning the factors associated with SRH in Iran are quite scarce. | Many thanks for your comments. It was conducted.  Page 4, lines 106-107. |
| **Methods** | |
| 1. I don't agree with the methods’ section structure. We recommend using the STROBE checklist for observational studies to report on the study, especially the methods section. | Many thanks for your comments. The method's section structure was revised based on the STROBE checklist. |
| 2. Line 116: The research should have one design, descriptive or analytic. In this case, the study is an analytic cross-sectional. The authors could simply write “cross-sectional study”. | Many thanks for your comments. It was revised. Page 1, line 2. Page 2 , line 41. |
| 3. Line 129: The authors should report the minimal sample size is 348, and report 350 in the results section. | Many thanks for your comments. It was revised. Page 1, line 41 and Page 5, line 116. |
| 4. Line 145: women included in this were aged 18 to 75 years. I can see a huge difference in age in this population, and I wonder if a 75-year-old woman can recall her daily life habits for physical activity, or if she can do physical activity. I think that this age interval is so large that it can affect the study result because of the difference in age characteristics. | The fact that the reviewer noted the good variety of age (18-75 years) of participants and the possibility of the differences between the memories of daily habits and physical activity among older women is insightful. The wide age bracket was chosen deliberately to get a rosy image of self-rated health in women within the community. Age was a covariate in crude and adjusted models of logistic regression, and its strong correlation with self-rated health was controlled. We acknowledge that this broad range can bring about heterogeneity and thus the future research can be stratified by organizing participants into smaller age groups in order to offer age specific information.  This limitation was added to the limitations section.  Page 13, lines 326-330. |
| 5. Are pregnant women included in the study? Pregnant women have a specific need for social support and adaptive physical activity. This should be specified in the inclusion and exclusion criteria. | Many thanks for your comments. Pregnant women were not included in this study and were an exclusion criterion. Being pregnant was added to the exclusion criteria in the Methods section.  Page 5, line 131. |
| 6. Line 152: Using two different data collection strategies (questionnaire for participants and interview for others) in the study creates information bias. How did the authors manage this? | Many thanks for your comments. The data collection method was not very different, and only in cases where participants were unable to complete the questionnaire for any reason, questions were asked by interviewers and then their responses were recorded confidentially. Hence, there is no possibility of bias.  More explanation added to the method section.  Page 6, lines 134-135. |
| 7. Line 174: DASS is a measurement tool for mental health status. “Mental health” is more appropriate than “psychological disorders.” | Many thanks for your comments. Based on the original version of the Depression Anxiety Stress Scales– 21 (DASS-21), this scale is 21-item self-report measure designed to assess the severity of general psychological distress and symptoms related to depression, anxiety, and stress in adults older adolescents (17 years +). So the phrase of “psychological disorders” in the title and other sections was edited.  Page 1, lines 1 and page 2, line 39 and page 7, line 173. |
| 8. Line 211-213: I do not agree with the authors to perform binary logistic regression should be performed because OR is applicable for case-control studies. However, linear logistic regression is correct and suitable for this study. We recommend performing a multivariable linear regression analysis or at least providing a reliable argumentation regarding the choice of logistic regression. | We sincerely thank the reviewer for this valuable comment regarding the choice of regression model. In our study, the dependent variable (self-rated health) was dichotomized into two categories (poor vs good SRH). For binary outcomes, logistic regression is widely recommended as the appropriate statistical method, since it estimates odds ratios to quantify the association between predictors and the likelihood of poor SRH. This approach is consistent with established statistical guidelines (Hosmer & Lemeshow, Applied Logistic Regression, 3rd edition).  Page 19, line 217. |
| **Results** | |
| 1. As mentioned above, I think that the age interval (18 to 75 years) is too large, which can affect the study results due to the difference in age characteristics (developmental patterns are different in each age category). The authors would rather present the data according to age groups (young and adult women (18 to 40 years, middle adulthood women 41 to 65 years, and old women aged more than 65 years). | We thank the reviewer for raising this point again. As noted in our response to the previous comment regarding the age variable, all analyses were performed accordingly to control for the confounding effect of age. We added this point as a limitation in our study. Please kindly refer to our earlier response for details.  Page 13, lines 326-330. |
| **Discussion** | |
| 1. Discussion is missing limitations of the study and its implications for practice. | Many thanks for your comments. The limitation section was added to the end of discussion.  Page 13, lines 326-330. |
| **Reviewer 3** | |
| 1. References used in the manuscript are outdated. I recommend updating the literature with more recent studies (3-5 years). | Many thanks for your comments. All references were revised and updated as much as possible. |
