# Supplementary material for: The influence of social support, physical activity, general psychological distress, and demographic characteristics on self-reported health status among women in Iran
Source: PLoS One. 2026 Feb 23;21(2):e0343015. doi: 10.1371/journal.pone.0343015 (PMC12928592; doi:10.1371/journal.pone.0343015)
Supplement: Attachment — Submitted filename: Reviewers Comments Response.2ndR.docx [file pone.0343015.s005.docx]

**Dear editorial board of *Journal of Plos One***

# Thanks for providing the comments of the respectful reviewer to us. We tried to revise the manuscript, titled “The Influence of Social Support, Physical Activity, General Psychological Distress, and Demographic Characteristics on Self-Reported Health Status among Women in Iran” based on the comments and respond it in the following table. Revisions has been shown as highlight in the manuscript. Hope the revisions are satisfactory now. However, we welcome any further constructive comments if required.

**Dr. Hadi Darvishigilan**

**Corresponding author**

| **Comments to the Author** | **Response** |
| --- | --- |
| 1. If the authors have adequately addressed your comments raised in a previous round of review and you feel that this manuscript is now acceptable for publication, you may indicate that here to bypass the “Comments to the Author” section, enter your conflict of interest statement in the “Confidential to Editor” section, and submit your "Accept" recommendation. | |
| **Reviewer 1:**  All comments have been addressed | Many thanks for your attention and time. |
| **Reviewer 2:**  All comments have been addressed | Many thanks for your attention and time. |
| 2. Is the manuscript technically sound, and do the data support the conclusions? | |
| **Reviewer 1:**  Yes**.** | Many thanks for your attention and time. |
| **Reviewer 2:**  Partly**.** | Many thanks for your comment. The conclusion section was revised. |
| 3. Has the statistical analysis been performed appropriately and rigorously? | |
| **Reviewer 1:**  Yes. | Many thanks for your attention and time. |
| **Reviewer 2:**   I Don't Know**.** | Many thanks for your comment. Considering that Dr. Shahab Rezaian (PhD in Epidemiology) has served as a statistical consultant and one of the study authors in various studies and has sufficient skills and expertise in this field, all statistical analysis of the study were performed and rechecked by him. Also, the other authors have sufficient skills and expertise in interpreting and analyzing the results, so there is no concern about the accuracy and precision of the statistical analysis. |
| 4. Have the authors made all data underlying the findings in their manuscript fully available? | |
| **Reviewer 1:**  Yes. | Many thanks for your attention and time. |
| **Reviewer 2:**   No. | Many thanks for your comment. As stated in the manuscript and in the submission system, all underlying study findings are available in the manuscript, and in the event of any ambiguity, the raw data of the study that has been analyzed and analyzed can be provided upon request. |
| 5. Is the manuscript presented in an intelligible fashion and written in Standard English? | |
| **Reviewer 1:**  Yes. | Many thanks for your attention and time. |
| **Reviewer 2:**  Yes**.** | Many thanks for your attention and time. |
| 6. Review Comments to the Author | |
| **Reviewer 1:** Yes.  We would like to thank the authors for addressing all comments. The manuscript now is more relevant and suitable for publication. | Many thanks for your attention and time. |
| **Reviewer 2:** Yes.  As previously noted, it is recommended that the references in this section be updated to primarily include sources published within the last 3–5 years, in order to better reflect the current state of knowledge. | Thanks for the comment from the respected referee. Older references have been updated. Currently, after updating the references, only references 29 and 31-41 are older, and given that these studies describe the initial design of the questionnaires used in this study in other contexts and their validity and reliability in Iran, it is not possible to update them. |
